# The small-molecule BMH-21 directly inhibits transcription elongation and DNA occupancy of RNA polymerase I *in vivo* and *in vitro*

**DOI:** 10.1016/j.jbc.2021.101450

**Published:** 2021-11-25

**Authors:** Ruth Q. Jacobs, Abigail K. Huffines, Marikki Laiho, David A. Schneider

**Affiliations:** 1 Department of Biochemistry and Molecular Genetics, University of Alabama at Birmingham, Birmingham, Alabama, 35294, USA; 2 Department of Radiation Oncology and Molecular Radiation Sciences and Sidney Kimmel Comprehensive Cancer Center, Johns Hopkins University School of Medicine, Baltimore, Maryland, USA

**Keywords:** cancer therapeutics, RNA polymerase I, transcription, rRNA, NET-seq, CF, core factor, ETS1, external transcribed spacer 1, ITS, internal transcribed spacer, K-S test, Kolmogorov–Smirnov test, mRNA, messenger RNA, NET-seq, native elongating transcript sequencing, Pol I, RNA polymerase I, rDNA, ribosomal DNA, rRNA, ribosomal RNA, TBP, TATA-binding protein

## Abstract

Cancer cells are dependent upon an abundance of ribosomes to maintain rapid cell growth and proliferation. The rate-limiting step of ribosome biogenesis is ribosomal RNA (rRNA) synthesis by RNA polymerase I (Pol I). Therefore, a goal of the cancer therapeutic field is to develop and characterize Pol I inhibitors. Here, we elucidate the mechanism of Pol I inhibition by a first-in-class small-molecule BMH-21. To characterize the effects of BMH-21 on Pol I transcription, we leveraged high-resolution *in vitro* transcription assays and *in vivo* native elongating transcript sequencing (NET-seq). We find that Pol I transcription initiation, promoter escape, and elongation are all inhibited by BMH-21 *in vitro*. In particular, the transcription elongation phase is highly sensitive to BMH-21 treatment, as it causes a decrease in transcription elongation rate and an increase in paused Pols on the ribosomal DNA (rDNA) template. *In vivo* NET-seq experiments complement these findings by revealing a reduction in Pol I occupancy on the template and an increase in sequence-specific pausing upstream of G-rich rDNA sequences after BMH-21 treatment. Collectively, these data reveal the mechanism of action of BMH-21, which is a critical step forward in the development of this compound and its derivatives for clinical use.

RNA polymerase I (Pol I) activity accounts for 60% of the transcriptional activity of a growing eukaryotic cell (1). Pol I is responsible for synthesizing the majority of the ribosomal RNA (rRNA) that assembles with ribosomal proteins to form the eukaryotic ribosome. Since Pol I transcription of the ribosomal DNA (rDNA) is the first, rate-limiting step of ribosome biogenesis (2, 3, 4), Pol I activity is directly proportional to the rates of cell growth and proliferation (5). In *Saccharomyces cerevisiae* (yeast) cells, Pol I transcribes the rDNA that is organized in approximately 200 tandem repeats on chromosome XII. Each 9.1 kb repeat contains the 35S gene, transcribed by Pol I, and the 5S gene, transcribed by Pol III. Transcription of the 35S gene by Pol I yields the precursor 35S rRNA that is co- and post-transcriptionally modified (6) to generate mature 18S, 5.8S, and 25S rRNAs. The rRNA synthesized by Pols I and III is processed and complexed with ribosomal proteins in the nucleolus. Finally, ribosomes are exported to the cytoplasm to execute protein synthesis.

In most cancers, Pol I transcription is upregulated and drives ribosome biogenesis and subsequent protein synthesis (7, 8, 9). High levels of protein synthesis are required to maintain robust cancer cell growth and proliferation (9, 10, 11). While ribosome biogenesis has been targeted to reduce cancer growth, most therapeutics fail to directly influence Pol I transcription (12, 13, 14). It is critical to discover direct inhibitors of Pol I transcription to minimize DNA damage (15) and to reduce interference with messenger RNA (mRNA) synthesis and translation (16). Therefore, Pol I transcription is an excellent target for selective inhibition of cancer cell growth (8, 17, 18, 19). Several Pol I transcription inhibitors have been investigated as anticancer therapeutics (20), including ML-246 (21) and CX-5461 (22, 23). CX-5461 was the first Pol I inhibitor to complete Phase I clinical trials. It is thought to inhibit Pol I transcription by competing with the preinitiation complex protein, SL1, for the rDNA promoter (22, 24). CX-5461 treatment resulted in a decrease of rDNA transcription in patients (25) and a reduction in cancer cell proliferation and tumor volume in mammalian cancer cell lines (26). In light of the progress of Pol I transcription-targeting anticancer therapeutics, there is a demand to discover and characterize highly specific Pol I-inhibiting therapeutic compounds.

A high-throughput cell-based screen revealed a novel p53-activating compound, BMH-21 (27), whose derivatives are currently in preclinical development. BMH-21 intercalates into GC-rich regions of the rDNA (28) to inhibit Pol I transcription without inducing DNA damage (29). BMH-21 treatment destabilizes the largest subunit of Pol I, A190 in yeast and RPA194 in mammals, triggering its degradation (29, 30, 31). Early studies found that BMH-21 displayed antiproliferative effects in tumor cell lines, *ex vivo* prostate tissue culture, and mouse models (27). The mechanism of BMH-21 inhibition of rRNA synthesis is not fully understood. It was previously demonstrated that BMH-21 inhibits Pol I transcription elongation (31) and reduces Pol I occupancy on the rDNA *in vivo* (30). The effects of BMH-21 on Pol I transcription and the degradation of A190/RPA194 are conserved between yeast and humans, making the yeast system an exceptional tool for defining BMH-21’s mechanism of action. Our aim was to provide a comprehensive analysis of BMH-21’s influence on Pol I catalyzed transcription.

In this study, we leveraged high-resolution *in vitro* and *in vivo* techniques to elucidate BMH-21’s mechanism of inhibition of Pol I using the yeast model system. We discovered that Pol I transcription initiation, promoter escape, elongation, and occupancy of the rDNA were inhibited by BMH-21. BMH-21 reduced Pol I’s processivity *in vitro*, evidenced by reduced full-length RNA product synthesis. Additionally, we found that Pol I transcription elongation is the most sensitive step to BMH-21 treatment. Our *in vitro* conclusions were complemented *in vivo* with native elongating transcript sequencing (NET-seq). Pol I occupancy of the rDNA was reduced after acute BMH-21 treatment and Pol I elongation complexes preferentially paused upstream of G-rich sequences. This study reveals the mechanism of action for this small-molecule inhibitor that is supported both *in vitro* and *in vivo*.

## Results

### Promoter-dependent *in vitro* transcription experiments probe the sensitivity of PoI I transcription steps to BMH-21 treatment

**Figure 1:**
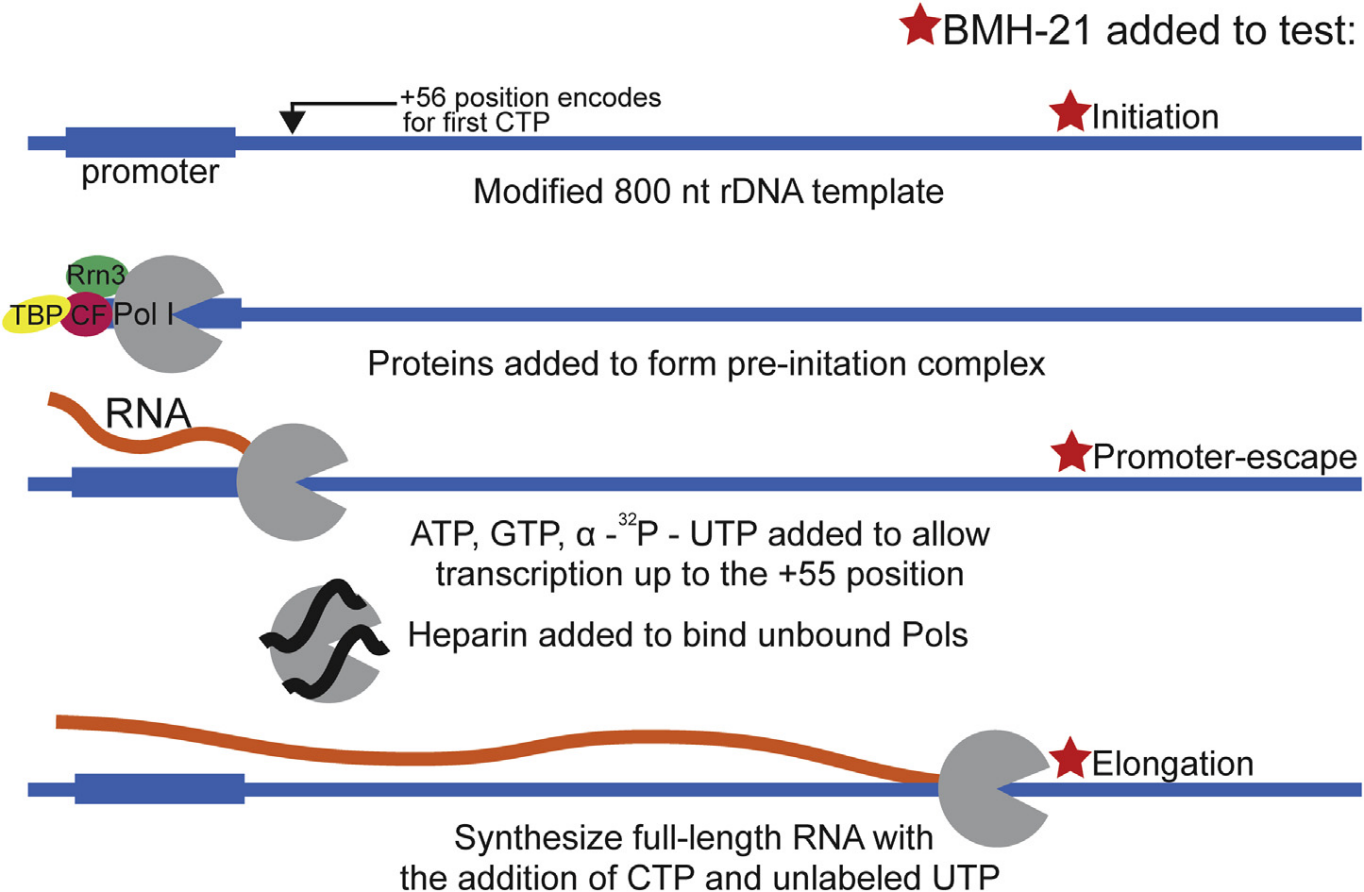
**Promoter-dependent *in vitro* transcription assay.** A modified rDNA template, which does not encode for CTP until the +56 position, is incubated with proteins required for Pol I transcription. NTPs, limiting for CTP, are provided to allow transcription up to the +55 position for 5 min. Heparin is added to prevent multiple rounds of transcription. Finally, CTP and unlabeled UTP are added to release Pols from the +55 position, allowing them to transcribe the remainder of the 800 nt template. BMH-21 is added to different steps illustrated in the schematic (*red stars*) to test effects on transcription initiation, promoter escape, and elongation. Pol I, RNA polymerase I.

To identify the impact of BMH-21 treatment on transcription catalyzed by Pol I, we utilized a fully reconstituted transcription assay (32) that includes all the required purified components for Pol I transcription: Pol I, core factor (CF), TATA-binding protein (TBP), and Rrn3 (Fig. 1). The template DNA includes a modified yeast rDNA template, mutated to not encode for C until the +56 position (with respect to the transcription start site). Once all the purified protein components are assembled, the DNA template and NTP substrates (ATP, GTP, α-^32^P-UTP) are added to allow the polymerase to initiate transcription and transcribe to the +55 position. Heparin is added to sequester unbound Pols to ensure we observe a single round of transcription. Finally, CTP and unlabeled UTP are added, releasing the polymerases from the +55 position to transcribe the remainder of the 800 base pair template. Reactions are run on polyacrylamide gels to resolve resultant RNA products for further analysis.

To determine the impact of BMH-21 on different steps in Pol I transcription, we added BMH-21 at different points in the reaction scheme. By varying the order of addition, these assays probe for the influence of BMH-21 on Pol I transcription initiation, promoter escape, and elongation (red stars in Fig. 1).

### Transcription initiation by Pol I is reduced in the presence of BMH-21

**Figure 2:**
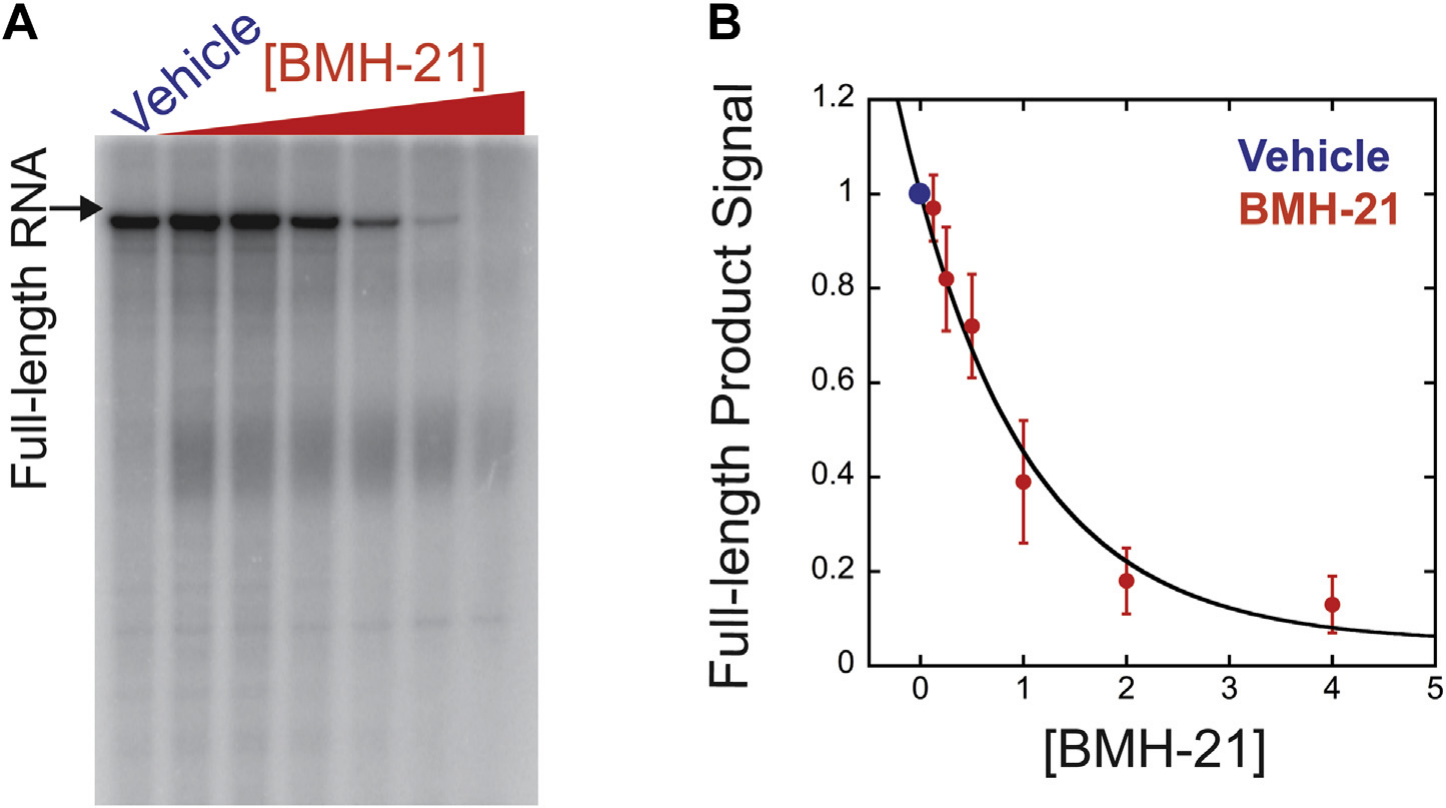
**BMH-21 inhibits early Pol I transcription initiation.***A*, vehicle or BMH-21 (125 nM–4 μM) was added to the rDNA template prior to preinitiation complex formation. Reactions proceeded for 10 min after CTP and unlabeled UTP were added. Full-length RNAs were resolved on polyacrylamide gels. *B*, plot of full-length RNA product signal over [BMH-21] treatment. Data were fit to a single exponential equation. Experiments were executed in triplicate and the mean is plotted with error bars corresponding to the standard deviation about the mean.

Transcription initiation involves many steps: first, transcription factors bind to the promoter region. Then, Pol I is recruited to form a preinitiation complex. DNA is unwound and initial RNA synthesis begins. The final stage of transcription initiation, promoter escape, occurs when Pol I clears the promoter region and advances on the template. To assay the impact of BMH-21 on transcription initiation, we added BMH-21 (125 nM–4 μM) or vehicle to the template prior to adding Pol I, TBP, CF, and Rrn3. ATP, GTP, and α-^32^P-UTP (20 μM) were provided for 5 min to allow Pol I to transcribe up to the +55 position. CTP and unlabeled UTP (20 μM) were added to the synchronized polymerases, releasing them from the +55 halt point. Each reaction proceeded for 10 min, allowing all complexes to synthesize full-length RNA products. Reactions were run on polyacrylamide gels and the abundance of full-length RNAs was analyzed (Fig. 2*A*). The amount of RNA synthesized by Pol I was reduced by increasing concentrations of BMH-21. Data were fit to a single exponential to calculate an IC_50_ value of 0.81 μM BMH-21 (Fig. 2*B*).

To control for the possibility that reduced full-length RNA products in the presence of BMH-21 (Fig. 2*A*) resulted from an inhibition of elongation rather than initiation, we treated Pol I transcription reactions during the elongation phase with vehicle or BMH-21 (1 μM) over a time course. Preinitiation complexes were incubated with ATP, GTP, and α-^32^P-UTP (20 μM) for 5 min. Simultaneously, BMH-21 (1 μM) or vehicle was added to the reactions with CTP and unlabeled UTP (20 μM). Reactions were stopped after 1, 4, and 10 min and run on polyacrylamide gels (Fig. S1). The time courses reveal that BMH-21 caused a delay in accumulation of full-length products, evident at the 4-min time points. However, the amplitude of the full-length products at the 10-min time points was identical between the vehicle- and BMH-21-treated samples (Fig. S1). Therefore, 10-min time points, as shown in Figure 2*A*, are not sensitive to transcription elongation rate effects. The simplest explanation for these data is that the reduction of full-length products in the presence of BMH-21, shown in Figure 2*A*, is due to inhibition of transcription initiation.

### BMH-21 disrupts Pol I clearing of the rDNA promoter

**Figure 3:**
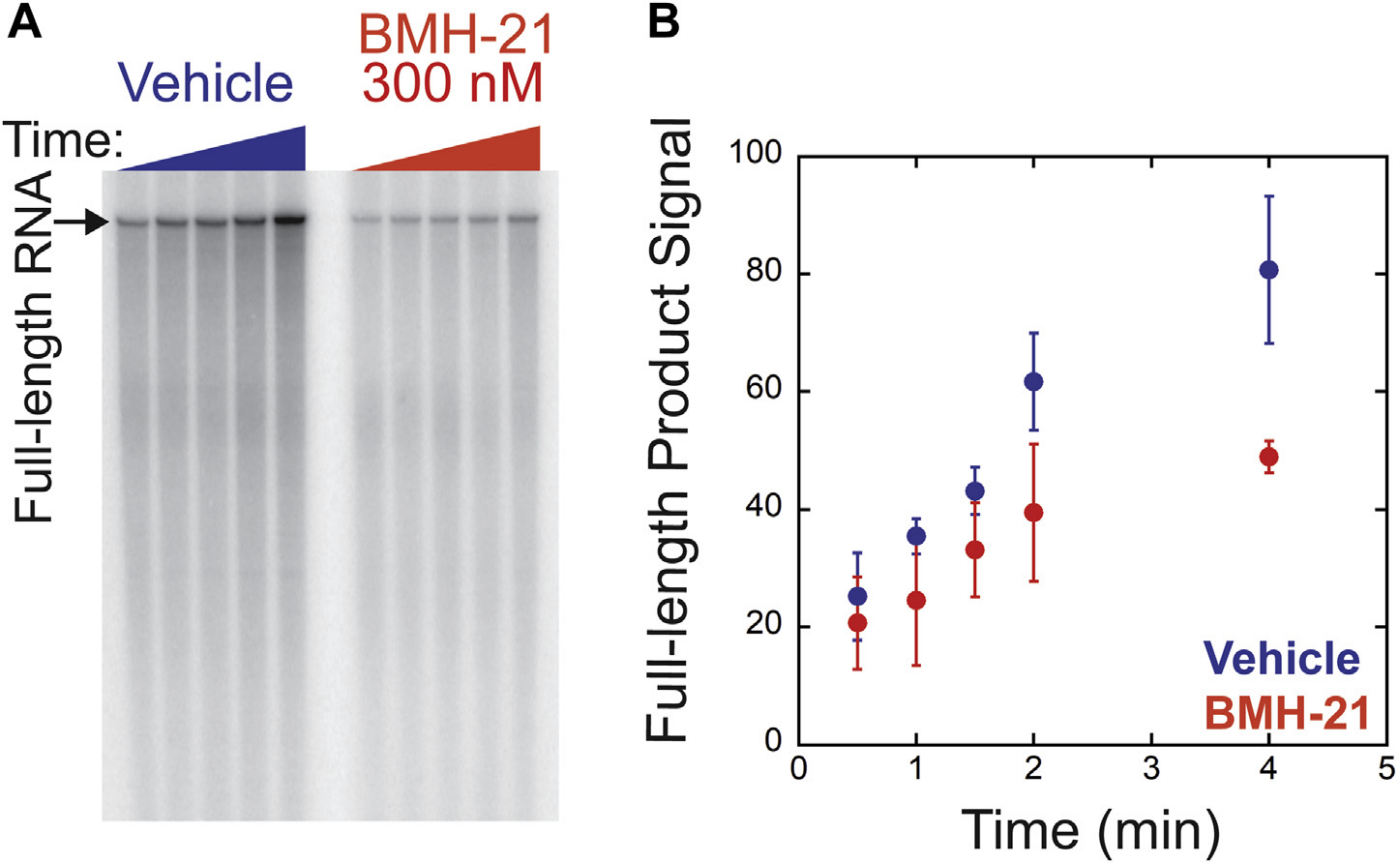
**BMH-21 reduces promoter-escape efficiency.***A*, Pol I transcription reactions were treated with vehicle or BMH-21 (300 nM) during the addition of ATP, GTP, and α-^32^P-CTP. Reactions were stopped at 0.5, 1, 1.5, 2, and 4 min after CTP and unlabeled UTP addition. Polyacrylamide gels resolved full-length RNAs. *B*, plot of full-length RNA product signal over time for vehicle and BMH-21 conditions. Experiments were executed in triplicate and the mean is plotted with error bars corresponding to the standard deviation about the mean. Pol I, RNA polymerase I.

We found a reduced amount of full-length RNA products when BMH-21 was added to the rDNA template, suggesting that BMH-21 inhibits transcription initiation. To determine if promoter escape was specifically inhibited by BMH-21, we formed the preinitiation complexes and added BMH-21 (300 nM) simultaneously with ATP, GTP, and α-^32^P-UTP (20 μM). Pol I complexes were allowed to synthesize through the promoter region up to the +55 position for 5 min. At t = 0, CTP and unlabeled UTP were added to the reactions and were stopped over time (30 s–4 min). The full-length RNAs were resolved on polyacrylamide gels (Fig. 3*A*).

If BMH-21 interfered with Pol I’s ability to clear the promoter and move down the template, we would detect a reduction in full-length RNAs. We found that Pol I synthesized less full-length products in the presence of BMH-21 compared with the vehicle control. We observed a suppression of full-length products over the time course in the presence of BMH-21, maximally causing a 49% reduction at 4 min (Fig. 3*B*). We conclude that multiple steps in transcription initiation are vulnerable to BMH-21.

### BMH-21 treatment robustly inhibits Pol I transcription elongation

Previously, we found that BMH-21 slows transcription elongation based on the observation that full-length RNA accumulation was slower in the presence of the compound (31). While strongly suggestive of direct inhibition of Pol I transcription elongation, this approach does not provide quantitative evaluation of transcription elongation kinetics. Here, we deploy a more quantitative assay (33) to directly measure Pol I transcription elongation rate in the presence of increasing concentrations of BMH-21.

**Figure 4:**
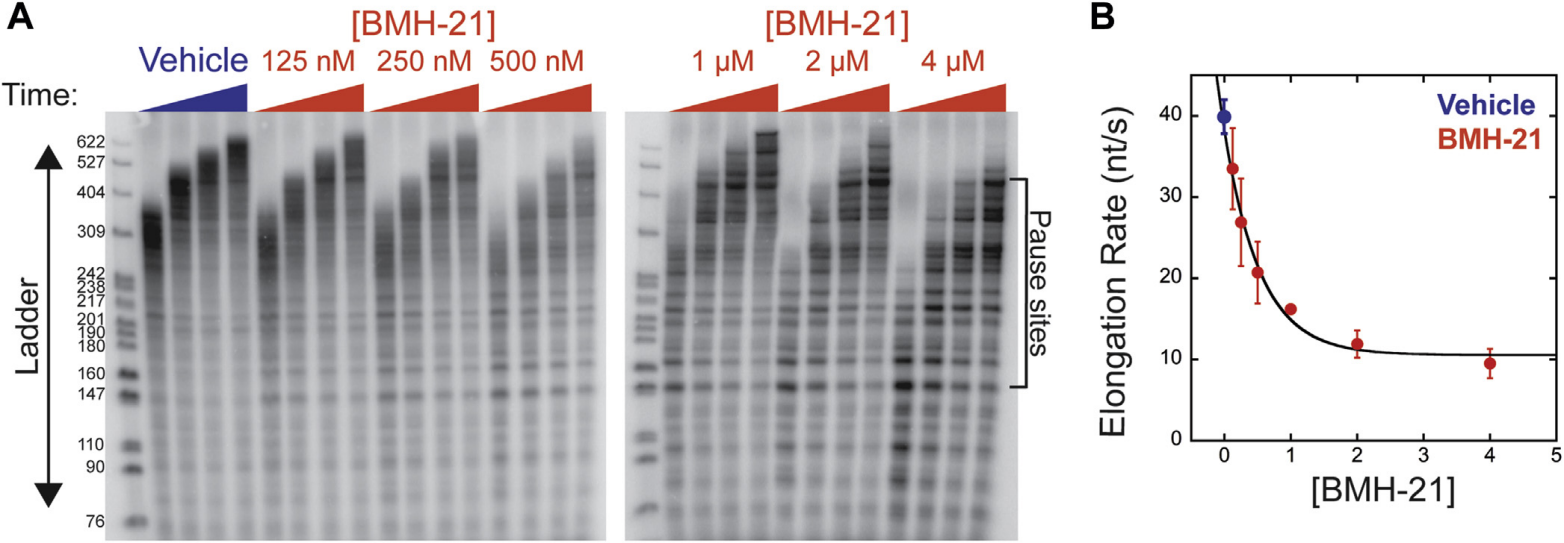
**BMH-21 decreases Pol I elongation rate and increases pausing.***A*, Pol I transcription reactions were treated with vehicle or BMH-21 (125 nM–4 μM) when CTP and unlabeled UTP were added. Reactions treated with vehicle or 125 to 500 nM BMH-21 were stopped at 4, 6, 8, and 10 s. Reactions treated with 1 to 4 μM BMH-21 were stopped at 5, 10, 15, and 20 s. Reactions were run on polyacrylamide gels alongside a nucleic acid ladder. *B*, elongation rate (nt/s) was plotted over [BMH-21] treatment. Data were fit to a single exponential equation. Experiments were executed in triplicate and the mean is plotted with error bars corresponding to the standard deviation about the mean. Pol I, RNA polymerase I.

Preinitiation complexes were formed and incubated with ATP, GTP, and α-^32^P-UTP (1 mM) for 5 min. At t = 0, CTP and unlabeled UTP (1 mM) were added simultaneously with BMH-21 or vehicle to release synchronized Pols from the +55 position. For each treatment condition (vehicle or 125 nM–4 μM BMH-21), four time points were collected. Reactions were resolved on polyacrylamide gels alongside a reference nucleic acid ladder to calculate the RNA length (nt) of the leading population of synthesized RNAs at each time point (Fig. 4*A*). Transcription elongation rate was measured by plotting the RNA length as a function of time and calculating the slope of the resultant line (Fig. S2). Transcription elongation rate was reduced by BMH-21 in a concentration-dependent manner with an IC_50_ of 0.43 ± 0.06 μM BMH-21 (Fig. 4*B*). Additionally, we visualized increased Pol I pausing in the BMH-21-treated reactions, evidenced by shorter RNA bands observed throughout the time courses, but this pausing was absent in the vehicle-treated reactions (Fig. 4*A*). Interestingly, the locations of the pause sites were conserved across the BMH-21 treated reactions. These data suggest that BMH-21 causes sequence-specific pausing of Pol I *in vitro*. Taken together, these data show that BMH-21 inhibits transcription initiation, promoter escape, and elongation (Figure 2, Figure 3, Figure 4).

### Vehicle and BMH-21-treated NET-seq libraries are highly reproducible

NET-seq probes for polymerase occupancy, which we define here as the positioning of Pol I on the rDNA template, at single-nucleotide resolution *in vivo.* This technique was first developed to study Pol II (34), but we recently adapted and optimized it to detect Pol I occupancy with high reproducibility (35, 36, 37). NET-seq for both vehicle- and BMH-21-treated yeast was performed in biological triplicate. The vehicle-treated libraries were aligned to the yeast genome and the 5′ read ends (which correspond to the 3′ ends of nascent transcripts) were plotted with respect to their position on the 35S gene (Fig. S3*A*, left panel). At each position, the “counts” value indicates the number of Pol I complexes occupying that position on the rDNA at the time of harvest. A high count value represents a highly occupied position or peak, whereas a low count value can be interpreted as a lesser occupied position or trough. While transcription elongation kinetics cannot be determined from these experiments, it is reasonable to equate peaks with positions where Pol I is stalled on the template, whereas troughs may represent regions of rapid transcription elongation by Pol I. To assess whether the Pol I occupancy patterns displayed for the vehicle-treated replicates were statistically similar, the Spearman correlation test was executed (Fig. S3*A*, right panel). This test ranks the occupancy in two replicates by count (highest to lowest), compares the ranking between the replicates, and generates a coefficient value. This value indicates the correlation between the two replicates, where 1 represents 100% similarity. The histogram generation and Spearman correlation test analysis were repeated for the BMH-21-treated samples (Fig. S3*B*), and like the vehicle-treated data, these results also displayed high similarity. Therefore, we concluded that this technique is highly reproducible and is a powerful method to visualize Pol I occupancy under treatment conditions at high resolution *in vivo*.

### BMH-21 reduces Pol I occupancy of the rDNA *in vivo*

**Figure 5:**
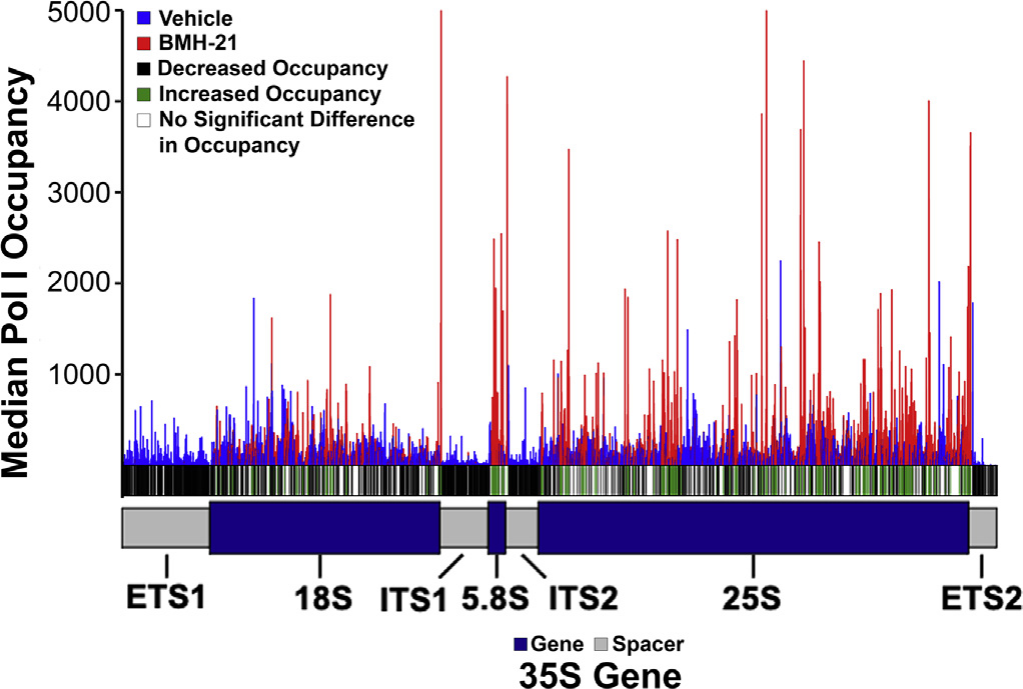
**Pol I occupancy was decreased after BMH-21 treatment.** The median Pol I occupancy for vehicle-treated (*blue*) and BMH-21-treated (*red*) libraries was plotted, and a *t*-test was executed at each position in the 35S gene. If the *p*-value was less than 0.05, the occupancy difference between treatment groups was deemed significant. Underneath the histogram, it was indicated whether occupancy was significantly increased (*black*), significantly decreased (*green*), or displayed no significant change (*white*) in the BMH-21-treated samples compared with the vehicle-treated. Pol I, RNA polymerase I.

Using NET-seq, we tested the effect of acute BMH-21 treatment (50 μM for 2 min) on Pol I occupancy *in vivo* as compared with vehicle treatment. We plotted the median Pol I occupancy across the three replicates for each treatment (Fig. 5). At each position, a *t*-test was executed to determine the *p*-value between treatments. A *p*-value < 0.05 was deemed a significant difference in occupancy and was denoted by either a black (decreased) or green (increased) bar below the histogram, for the BMH-21-treated samples with respect to vehicle-treated. These data indicate that there is a significantly different occupancy pattern between the two treatment groups, as there is a depletion of Pol I especially in the spacer regions [external transcribed spacer 1 (ETS1), internal transcribed spacer 1 (ITS1), internal transcribed spacer 2 (ITS2), external transcribed spacer 2 (ETS2)].

**Figure 6:**
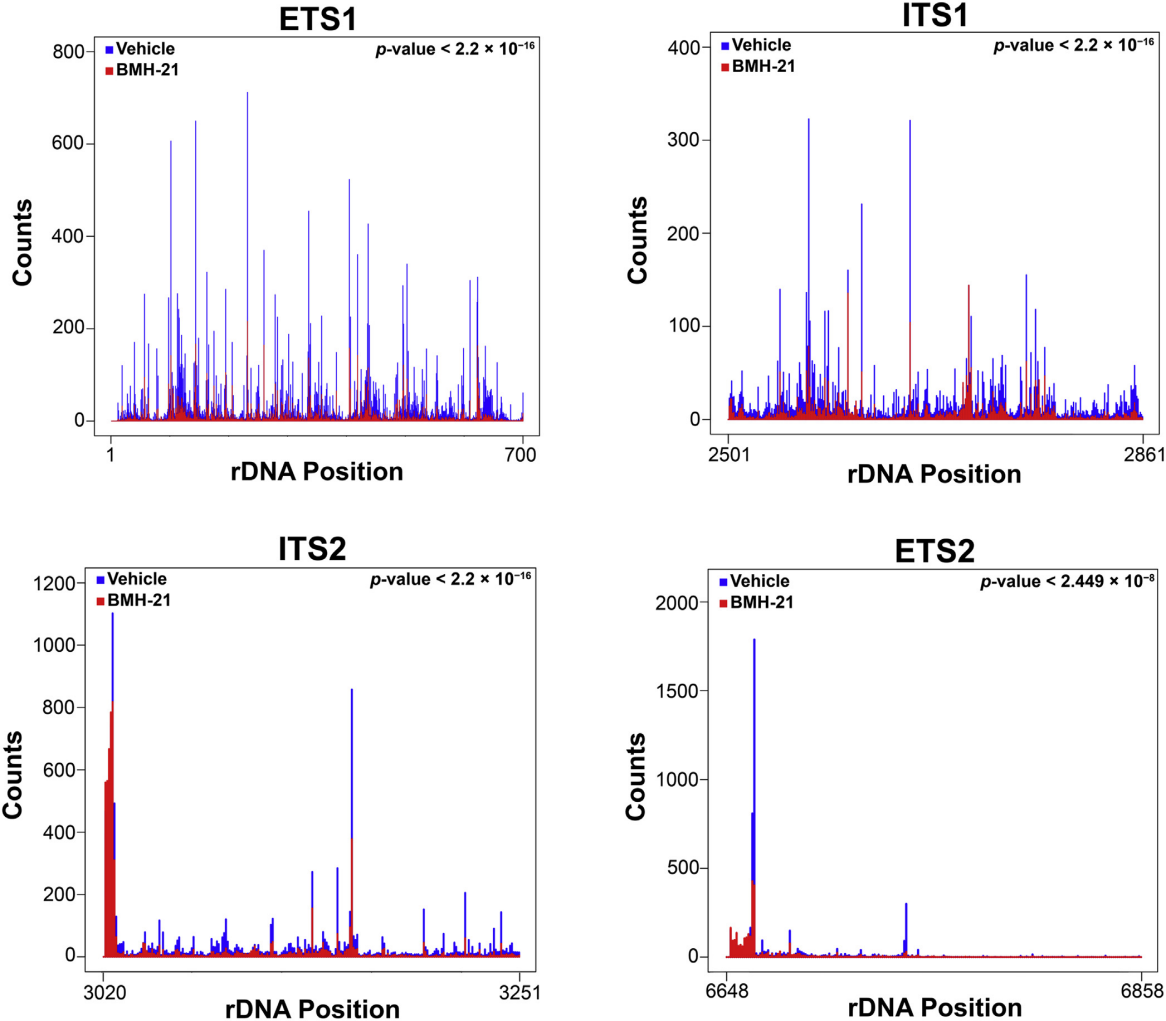
**Pol I occupancy patterns in spacer regions validate reduced occupancy after BMH-21 treatment gene-wide.** The median Pol I occupancy was plotted for vehicle-treated (*blue*) and BMH-21-treated (*red*) libraries in each spacer region. The K-S test was performed to determine whether the overall distribution of occupancy in each spacer region was significantly different between the two treatment conditions. The *p*-value determined by that test is included in the *top right* corner of each graph. Pol I, RNA polymerase I.

One limitation of the NET-seq technique is that there is unavoidable mature rRNA contamination in the libraries. This is especially evident in Figure 5, where there are still Pol I peaks present in the gene regions of the BMH-21-treated samples, despite the reduction in occupancy in the spacer regions. From previous literature, it has been established that processing/maturation of the rRNA begins to occur co-transcriptionally and results in removal of the spacer RNAs (32, 38, 39, 40). Furthermore, we performed NET-seq using a strain of yeast containing untagged Pol I to investigate the pattern of contamination in our samples. The data from this untagged Pol I control demonstrate that while there is high signal in the gene regions, reads mapping to the spacer regions were minimal (Fig. S4), confirming that the contamination in our sample is, indeed, mature rRNAs. Therefore, since it is impossible to remove mature rRNA contamination in the experiment, the spacer regions are critically important for observing nascent transcription by Pol I. Thus, we performed an in-depth analysis of Pol I occupancy in these regions (Fig. 6). In all four of the rDNA spacer regions, there was robust reduction in Pol I occupancy in the BMH-21-treated samples as compared with vehicle-treated. We employed the Kolmogorov–Smirnov test (K-S test) to evaluate the significance of this altered distribution between treatment groups within these regions and found these differences to be significant (indicated by the *p*-values in Fig. 6). These data are consistent with the patterns observed in Figure 5 and reveal that BMH-21 treatment significantly reduces Pol I occupancy on the rDNA in comparison to vehicle treatment even after only 2 min. In combination with the *in vitro* results shown in Figures 2 and 3, this observation suggests that BMH-21 inhibits transcription initiation by Pol I.

### BMH-21 induces Pol I pausing upstream of G-rich rDNA sequences

**Figure 7:**
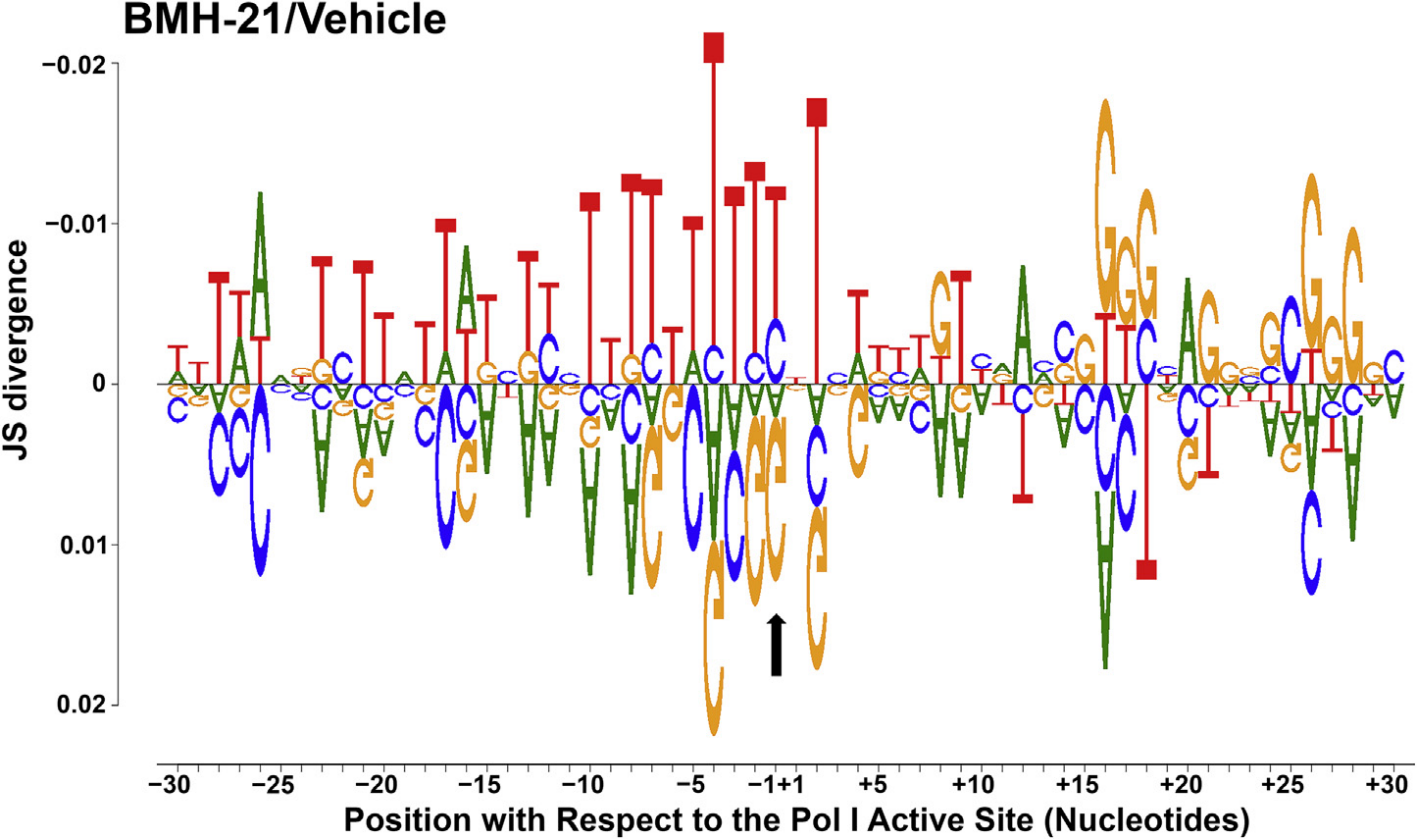
**Pol I is stalled upstream of particularly G-rich regions of the rDNA spacer regions.** This figure was created using the DiffLogo program (version 2.14.0) and displays sequences both *upstream* and *downstream* of Pol I for the top 2.5% occupied positions in the spacer regions only (ETS1, ITS1, ITS2, and ETS2). The DiffLogo for the BMH-21-treated samples is plotted on top and displays the sequences that are enriched with comparison to the vehicle-treated libraries. In contrast, the DiffLogo for the vehicle-treated samples is displayed underneath and indicates the enriched sequences with comparison to the BMH-21-treated samples. The *black arrow* identifies the last incorporated nucleotide in the nascent transcript. ETS, external transcribed spacer; ITS, internal transcribed spacer; Pol I, RNA polymerase I.

We found that there was a reduction in overall occupancy of Pol I across the 35S gene, which suggests that BMH-21 treatment inhibits transcription initiation. To evaluate potential effects on transcription elongation, we compared *in vivo* pausing after either vehicle- or BMH-21-treatment by examining the sequence context of the top 2.5% most occupied positions (consistent with previously published findings (35, 36, 37)) in the spacer regions only. The DiffLogo plot in Figure 7 displays the BMH-21-treatment logo on top and the vehicle treatment logo underneath, with the black arrow indicating the position of the last incorporated nucleotide into the nascent transcript. We observed that in the BMH-21-treated samples, Pol I is frequently paused in T-rich regions of the rDNA that are directly upstream from G-rich regions. The opposite pattern is true in the vehicle-treated samples, where Pol I is stalled in G-rich regions of the template. This finding is consistent with previous literature suggesting that BMH-21 intercalates into GC-rich regions of DNA (30). We hypothesize that upon intercalation of BMH-21 directly downstream of the elongation complex, this could induce added torsional stress, causing Pol I to be halted on the template. Furthermore, these data are consistent with *in vitro* results (Fig. 4) that revealed sequence-specific effects by BMH-21 on transcriptional pausing. Thus, BMH-21 has direct negative effects on transcription initiation and elongation by Pol I *in vitro* and *in vivo*.

## Discussion

### BMH-21 directly inhibits Pol I transcription and causes a decrease in rRNA synthesis by reducing Pol I occupancy of the rDNA

The data presented here, resulting from high-resolution *in vitro* and *in vivo* experiments, elucidate BMH-21’s mechanism of inhibition of transcription by Pol I. We showed that BMH-21 added during three transcription steps inhibited Pol I transcription through a decrease in full-length RNAs and elongation rate and an increase in amplitude of short, incomplete RNAs. Despite sensitivity to BMH-21 during all of the transcription steps tested, we conclude that the transcription elongation phase is most vulnerable to BMH-21 inhibition based on the IC_50_ values calculated (Figs. 2 and 4). Altogether, these *in vitro* observations suggest that BMH-21 treatment reduces the amount of Pol I transcription complexes that are capable of efficient and processive transcription. These findings were complemented by our *in vivo* NET-seq experiments. We found that Pol I occupancy of the rDNA was reduced across the 35S gene (Fig. 5). Specifically, there was a significant decrease in occupancy in the spacer regions of the 35S gene, which serve as a reporter for nascent transcripts, since these regions are removed prior to maturation of the rRNAs (Fig. 6). In the BMH-21-treated samples, Pol I was preferentially paused upstream of G-rich rDNA regions (Fig. 7). These findings suggest that the intercalation of BMH-21 into the GC-rich regions of the rDNA introduces a barrier to Pol I elongation complexes. In contrast, T-rich regions of the rDNA were enriched within the RNA:DNA hybrid and upstream of the polymerase. Furthermore, NET-seq was repeated to test a longer BMH-21 treatment time of 16 min (Figs. S5–S8), and these results were consistent with the 2-min treatment findings (Figure 5, Figure 6, Figure 7). Taken together, our *in vitro* and *in vivo* analyses are complementary, providing new insight into BMH-21’s mechanism of action.

### BMH-21 influences Pol I activity similarly to other DNA intercalators

Our BMH-21 *in vitro* and *in vivo* results are consistent with the mechanism of action of other GC-intercalators including actinomycin, ethidium bromide, and proflavine (41, 42, 43). The Panov research group executed an extensive study of DNA intercalators using *in vitro* and cell-based techniques (41). They used a plasmid DNA template containing the rDNA promoter, very similar to our *in vitro* system. Consistent with our findings in Figures 2 and 3, early transcriptional events catalyzed by Pol I (initiation, promoter escape) are sensitive to DNA intercalators. Our *in vitro* IC_50_ concentration for transcription initiation by BMH-21, 810 nM, was within the range of IC_50_ values of proflavine, 9HE, Acridine Orange, and Thionine (41). Additionally, they found that Pol I transcription elongation is sensitive to DNA intercalators by measuring rRNA synthesis in human cells. Our IC_50_ measurement in Figure 4 revealed that BMH-21 is comparatively potent for inhibition of transcription elongation. Finally, DNA intercalators have been found to reduce Pol I occupancy similar to our results in Figures 5 and 6 (41). Taken together, our data suggest that BMH-21 acts similarly to other DNA intercalators by inhibiting early Pol I transcriptional events and Pol I occupancy of the rDNA.

### BMH-21 and CX-5461 inhibit ribosome biogenesis through different mechanisms

The first Pol I inhibitor to complete Phase I clinical trials was CX-5461 (22, 23). It was found to inhibit Pol I transcription by interfering with transcription initiation. It binds SL1, comprised of TBP and TBP-associated factors, to disrupt the release of the Pol I-Rrn3 complex from the rDNA promoter (22, 44). *In vivo* studies showed promising anticancer effects as treatment with CX-5461 decreased cancer cell and tumor viability in human cancer cell lines and murine xenografts (45, 46, 47, 48). Despite the establishment of CX-5461 as a direct and selective inhibitor of Pol I transcription, a recent study found that CX-5461 may stabilize G-quadruplex DNA and cause DNA damage (49). This finding cast doubt on the previous classification of CX-5461 as a direct Pol I inhibitor. Considering the controversy, an in-depth, multidimensional investigation of the mechanism of CX-5461 found that it did not induce cell death via interference with Pol I function, rather, it accomplished its cytotoxic effects *via* topoisomerase II (Top2) poisoning (50). Thus, the mechanism of action for CX-5461 remains somewhat controversial, despite its initial promise in the clinic. Clarity on this will be required to facilitate selection of patients most responsive to its therapeutic effects.

The mechanism of Pol I inhibition by the BMH-21 class of molecules is significantly different than that of CX-5461. BMH-21 intercalates into GC-rich DNA without causing DNA lesions (27, 29, 30). Unlike CX-5461, multiple Pol I transcription steps (initiation, promoter escape, and elongation) are inhibited by BMH-21. BMH-21 inhibits Pol I transcription through binding to the highly GC-rich content of the rDNA, presumably without directly associating with Pol I transcription machinery. Previous studies with BMH-21 suggest a direct negative effect on Pol I transcription (29, 30, 31). Here, we define the mechanism of action for the compound using biochemical and genomic approaches. While these results do not exclude the possibility that BMH-21 may affect other processes, these findings clearly reveal acute, direct, and potent effects of the compound on Pol I.

### How is BMH-21 specific to Pol I?

BMH-21 inhibition may be specific to Pol I transcription based on the chromatin organization of the rDNA. Our *in vitro* transcription DNA template lacks nucleosomes; therefore, it may be similar to the rDNA transcribed by Pol I *in vivo*. Unlike chromatinized genes transcribed by Pol II (51, 52), active rDNA repeats transcribed by Pol I lack ordered nucleosomes (28, 53, 54, 55, 56). Thus, based on BMH-21’s affinity for open, GC-rich rDNA, it could selectively impact Pol I transcription. Alternatively, it is possible that BMH-21 may bind many genomic loci, including loci transcribed by Pols II and III. Perhaps Pols II and III exhibit enzymatic features that render them insensitive to inhibition by BMH-21. We showed that Pols I and II possess unique biochemical properties (57). Nucleotide addition catalyzed by Pol I is significantly faster than by Pol II. Coupled with Pol I’s rapid elongation kinetics, it forms a less stable transcription elongation complex compared with Pol II. In contrast, Pol II nucleotide addition kinetics are significantly slower, and Pol II forms a very stable elongation complex. Pol I’s fast nucleotide addition kinetics and less stable elongation complexes may render Pol I specifically vulnerable to BMH-21 intercalation sites compared with Pol II. Determining whether BMH-21 selectively inhibits Pol I, compared with the other RNA polymerases, is a topic of ongoing investigation.

In conclusion, complementary *in vitro* and *in vivo* experiments demonstrate that BMH-21 inhibits Pol I transcription by reducing the efficiency of Pol I transcription initiation and elongation. Mechanistic studies are ongoing to elucidate the impact of BMH-21 on the kinetic mechanism of nucleotide addition by Pol I in the presence of BMH-21.

## Experimental procedures

### Yeast strain used for NET-seq experiments

Wild-type (WT) yeast (ade2-1 ura3-1 trp1-1 leu2-3, 112 his3-11,15 can1-100 RPA135-(HA)3- (His)7::TRP1m × 6 rpa190Δ::HIS3M × 6 carrying pRS315-RPA190) was used for these experiments. This strain was used previously to generate NET-seq data for Pol I occupancy in other studies (35, 36). To perform NET-seq for this study, cells were cultured at 30 °C with nutation. A yeast strain containing untagged Pol I (ade2-1 ura3-1 trp1-1 leu2-3, 112 his3-11,15 can1-100) was used to generate the NET-seq data in Figure S4.

### Purified proteins used within *in vitro* transcription assays

Pol I was purified from a protease-deficient yeast strain (58) with the largest subunit, A190, tagged with ten histidine residues. CF, Rrn3, and TBP were cloned into overexpression vectors and transformed into BL21(DE3)-pRosetta-2 *E. coli* cells. Purifications were performed as previously published (36, 59, 60).

### Promoter-dependent *in vitro* transcription assay

*In vitro* transcription experiments were performed as previously described (59). The purification of the modified rDNA template (C-less until +56 position) and protein components were confirmed with agarose and SDS-PAGE gels, respectively. Template DNA was incubated with Pol I, CF, TBP, and Rrn3 to allow for preinitiation complex formation. ATP, GTP, and α-^32^P-UTP were added to allow transcription until the +55 halt point for 5 min. Heparin is added to ensure a single round of transcription by binding unbound Pol I’s. CTP and unlabeled UTP were added to release the Pols from the +55 position, allowing for the synthesis of full-length RNA products. Reactions were stopped with the addition of 1 M ammonium acetate in 100% ethanol. RNA was precipitated overnight at −20 °C and then centrifuged at 13,000*g* for 10 min. Supernatant was discarded and the RNA pellets were resuspended in RNA loading dye. Samples were boiled and loaded on a 5% polyacrylamide (29:1, acrylamide to bis), 1 X TBE, 7 M urea gel. Gels were vacuumed dry and exposed in a phosphorimager cassette overnight. The phosphorimager screen was imaged by an Amersham Typhoon scanner, and the image was analyzed using ImageQuant.

### Determination of BMH-21 concentration for promoter-escape experiments

We originally treated Pol I transcription reactions during promoter escape with the IC_50_ calculated from transcription initiation experiments (Fig. 2). Due to low ratio of signal to background, we were unable to execute subsequent experiments at ∼800 nM BMH-21. To determine a BMH-21 concentration that would allow for quantifiable and reproducible signal, we tested different concentrations of BMH-21, 100 to 1600 nM. We selected 300 nM BMH-21 for experiments shown in Figure 3.

### Elongation rate determination

Quantification of Pol I transcription elongation rate was executed similarly as previously published (33). Gel images from transcription elongation experiments were analyzed in ImageQuant. Lanes are boxed within columns, each encompassing one reaction collected at a certain time point. The leading population RNAs are boxed and the average intensity point within the box is calculated. The average intensity point is correlated to the ladder to calculate the RNA length for each time point. Elongation rate is calculated by plotting RNA length (nt) over time (s) per condition and fitting to a line (Fig. S2).

### NET-seq experiments

NET-seq was performed based on an adaptation from previously described methods (35, 36, 37). Yeast was cultured the same as in previous literature (36), except that this adapted method required only 1 l of yeast per replicate, compared with the previous 3 l per replicate. Once cells reached an OD_600_ of 0.3, they were treated with either 50 μM of BMH-21 (for the BMH-21 samples) or an equivalent volume of the vehicle buffer (0.1 M NaH_2_PO_4_, pH 6; for the vehicle samples). BMH-21 (12H-Benzo[g]pyrido[2,1-b]quinazoline-4- carboxamide, N-[2(dimethylamino)ethyl]-12-oxo) was verified for purity using LC/MS mass spectrometry and ^1^H-NMR as previously published (31). Immediately following treatment, cells were placed back in the incubator to culture for an additional 1 min (for the 2-min experiment, Figure 5, Figure 6, Figure 7 and S3) or 15 min (for the 16-min experiment, Figs. S5–S8). Cells were harvested using an ∼1 min method and lysis was performed, both described previously (36).

To prepare for immunoprecipitation (IP), lysis buffer (20 mM Tris-HCl pH 7.9, 0.4% Triton X-100, 0.1% NP-40, 100 mM NH_4_Cl, 5 mM EDTA-Na pH 8.5, 1x HALT Protease Inhibitor, 25 U/ml SUPERase-in RNAse Inhibitor) (36) and blocking buffer (same as lysis buffer, with 10 mg/ml BSA added) were prepared on ice. Pierce anti-HA beads for each sample (equivalent to 4% of the lysis buffer required based on grindate weight of sample (36)) were washed three times at 4 °C with 1 ml lysis buffer and nutation. After the final wash, lysis buffer was discarded, and 500 μl blocking buffer was added to each bead-containing tubes. Beads were blocked overnight at 4 °C with nutation. IP, RNA extraction, and precipitation were performed as previously described (35, 36) except that 2 mg/ml BSA was added to the wash buffer, and bead washing was eliminated during the IP.

Following precipitation, samples were centrifuged, and linker ligation was performed the same as previously described (35, 36), except that RNA pellets were dissolved in 10 mM Tris-HCl, pH 6.9, and 20 U/μl SUPERase-in RNAse inhibitor was added the ligation mix. A DNA linker containing a unique molecular identifier was used (35). After linker ligation was complete, an enzymatic digestion of the excess linker was performed instead of a gel extraction using the following steps. First, 2 μl of 5′ deadenylase (NEB) was added to each sample and mixed thoroughly. Samples were incubated at 30 °C for 45 min. Next, samples were diluted 2.5X with water supplemented with 0.6X NEBuffer 2, and 2 μl of RecJ_f_ (NEB) was added and mixed well. Samples were incubated at 37 °C for 45 min. Then, 2.2 μl zinc fragmentation buffer (100 mM Tris-HCl, pH 7.0, 100 mM ZnCl_2_) (35) was added to each sample, and samples were incubated at 70 °C for 16 min. Finally, 2.5 μl 200 mM EDTA, pH 8.5, 1 μl glycoblue, and 360 μl ammonium acetate precipitation solution were added, and samples were precipitated for at least 2 h at −80 °C.

Samples were centrifuged, pellets were washed, and reverse transcription was performed as previously described (35, 36), except that reactions were incubated at 45 °C (instead of the previous 50 °C) for 30 min. A 10% polyacrylamide gel was prepared and pre-run, samples were loaded, and gel was run as previously described (36). Slices between approximately 120 and 600 nucleotides were excised from the gel and pulverized for each sample. Then, 500 μl water was added and gel slurries were incubated at −80 °C for 15 min and then at 70 °C for 15 min. Finally, slurries were placed at 30 °C with nutation overnight. The following day, each slurry was transferred to a Costar Spin-X Centrifuge Tube Filters (Corning), and tubes were centrifuged at 16,000*g* for 3 min at room temperature. The flow-through from each tube was transferred to a new 1.5 ml tube, and 32 μl 3 M NaCl, 940 μl 100% isopropanol, and 1 μl glycoblue were added to each sample. Samples were precipitated for at least 2 h at −80 °C.

Samples were centrifuged at 16,000*g* for 1 h at 4 °C and then washed as previously described (35, 36). Pellets were resuspended in 15 μl of 10 mM Tris-OAc, pH 7.9. Circularization, amplification, sample preparation/purification, and sequencing were performed as previously described (35, 36). Amplification primers for each sample are included in Table S1.

### NET-seq data analysis and statistical analysis

The data analysis pipeline used was identical to a previous publication (35).

Software and the versions used for these studies are included in Table S2. Reads were aligned to the *S. cerevisiae* genome assembly R64-1-1. The data used to generate the figures in this study have been deposited into NCBI’s Gene Expression Omnibus (61) and are available through the series accession number GSE175553.

The Spearman correlation test was used to detect reproducibility between replicates, and these data are included in the right panel of Figures S3 and S5. For this test, n = 2, where n is the number of replicates being compared for similarity. The K-S test was used to determine any significant differences in occupancy distribution between treatment groups. The results from the K-S test are shown in the inset in the top right corner of the graphs in Figures 6 and S7.

## Data availability

The datasets generated during this study are available at: GSE175553 on the NCBI GEO database. Further information and requests for resources and reagents should be directed to and will be fulfilled by the lead contact, David Schneider (dschneid@uab.edu).

## Supporting information

This article contains supporting information.

## Conflict of interest

The authors declare that they have no conflicts of interest with the contents of this article.
