# Supplementary material for: The small-molecule BMH-21 directly inhibits transcription elongation and DNA occupancy of RNA polymerase I in vivo and in vitro
Source: J Biol Chem. 2021 Nov 25;298(1):101450. doi: 10.1016/j.jbc.2021.101450 (PMC8683726; doi:10.1016/j.jbc.2021.101450)
Supplement: Figures S1–S8 and Tables S1 and S2 [file mmc1.docx]

**SUPPLEMENTAL INFORMATION FOR:**

**“The small molecule BMH-21 directly inhibits transcription elongation and DNA occupancy of RNA polymerase I *in vivo* and *in vitro”***

**
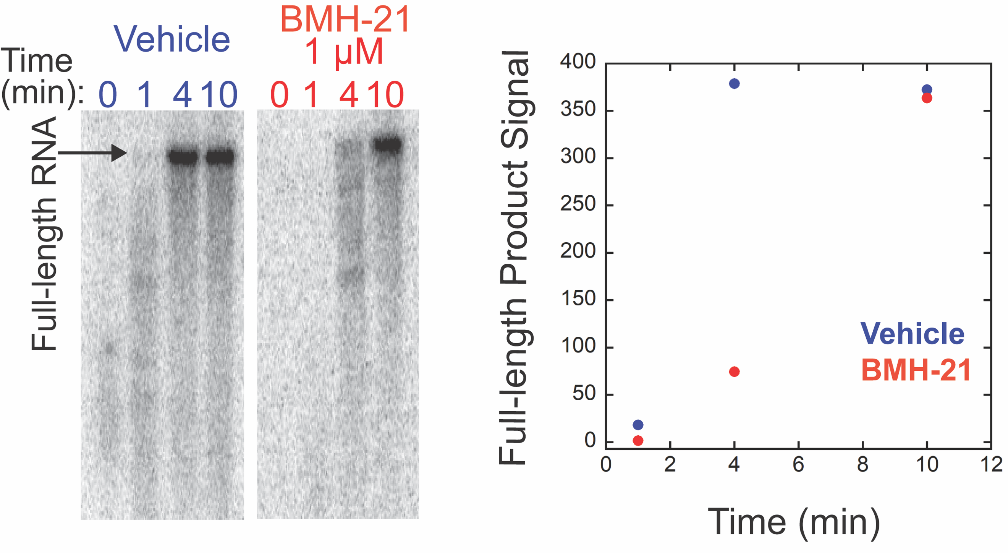
**

**Supplemental Figure S1. Ten-minute time points are not sensitive to elongation effects.** Vehicle or BMH-21 (1 µM) was added simultaneously with CTP to treat the elongation phase of Pol I transcription. Reactions were stopped at 1, 4, and 10 minutes. Synthesized RNAs were resolved on polyacrylamide gels. We plotted the full-length RNA product signal over time in the presence of vehicle or BMH-21 (1 µM). Experiment was executed once per treatment condition.

**
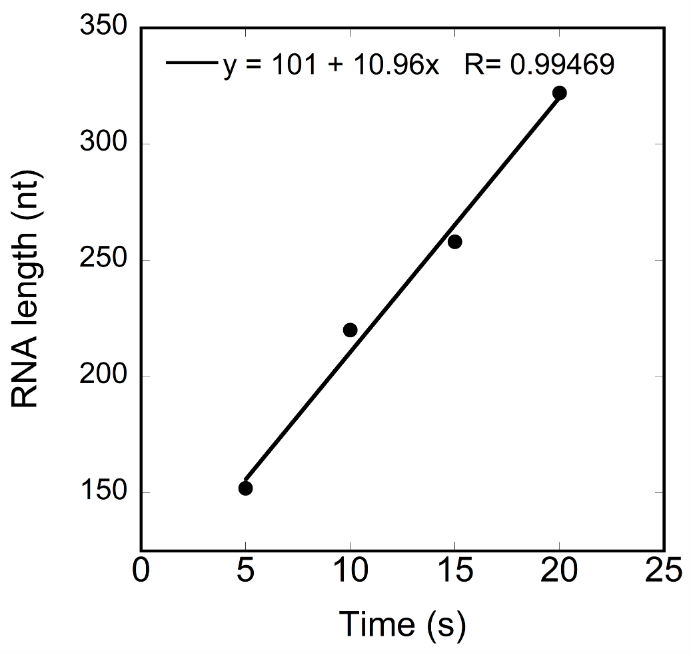
**

**Supplemental Figure S2. Elongation rate determined by fitting RNA length over time to a line.**

The length of RNA of the leading smear of RNAs was determined per time point based on the nucleic acid ladder in Figure 4. The length of RNA was plotted over time for each of the four time points per treatment condition. Data points were fit to a line and the slope was measured as the elongation rate in nucleotides per second.

**
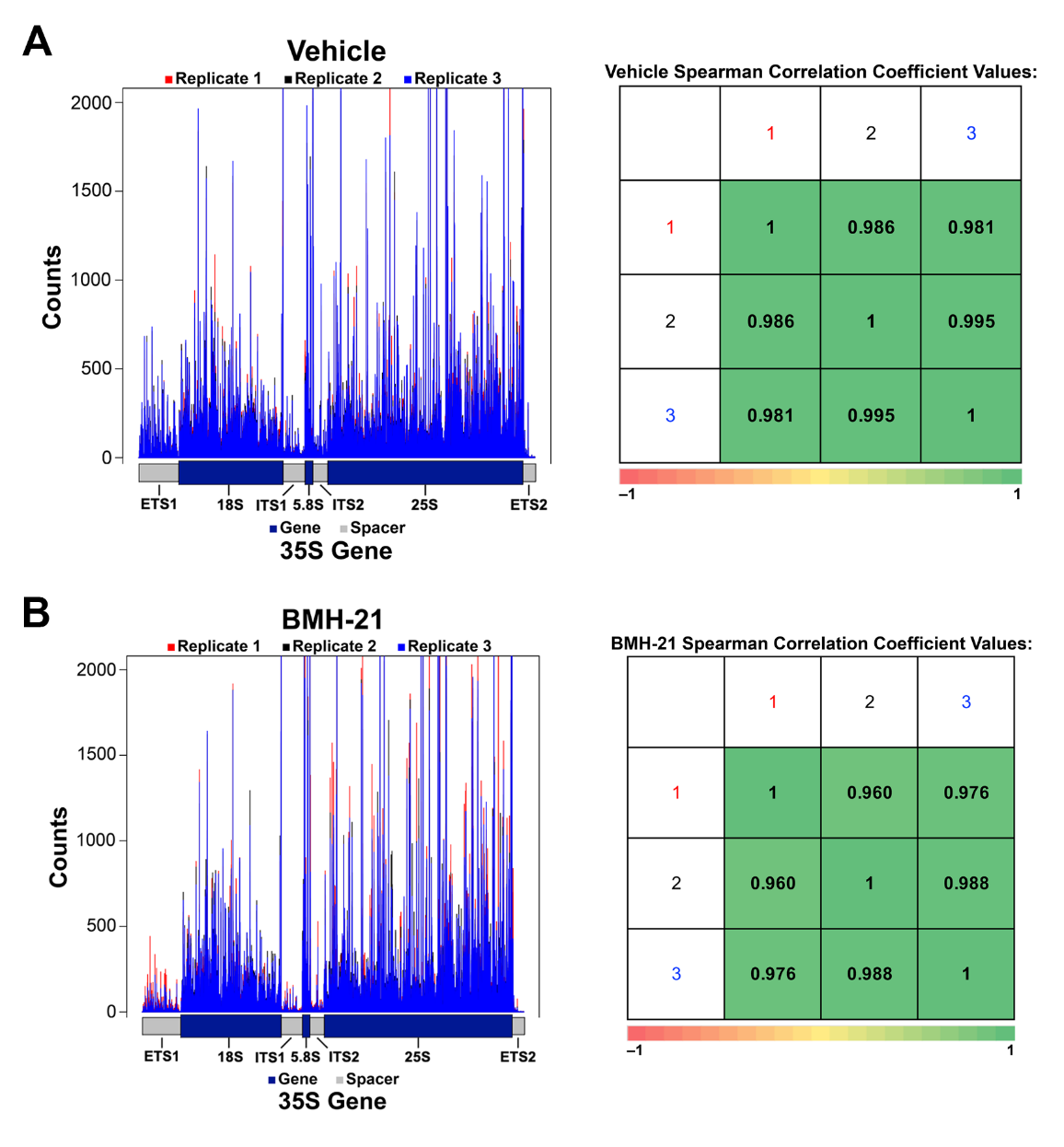
**

**Supplemental Figure S3. NET-seq experiments are reproducible.**

Yeast were treated for 2 minutes with either vehicle buffer (A) or BMH-21 (B), harvested, and prepared for NET-seq library generation. Histograms depicting Pol I occupancy (left panels) and Spearman correlation coefficient values (right panels) were plotted for vehicle-treated and BMH-21-treated yeast. These experiments were performed in triplicate, resultant libraries were aligned to the yeast genome (assembly R64-1-1), and 5’ read ends were mapped to the 35S gene.

**
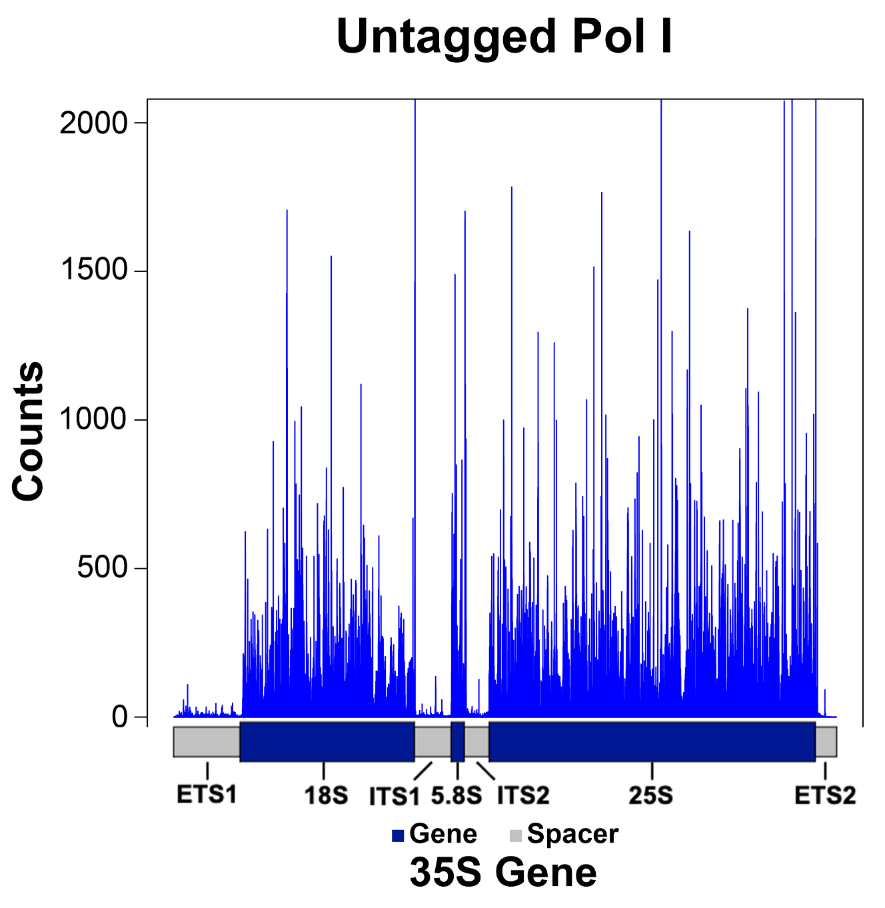
**

**Supplemental Figure S4. NET-seq performed with a yeast strain containing an untagged Pol I demonstrates a low amount of mature rRNA contamination in the spacer regions.**

NET-seq was performed with one sample of a yeast strain containing untagged Pol I. The 5’ end of these resultant reads were mapped back to the yeast genome and plotted in a histogram.

**
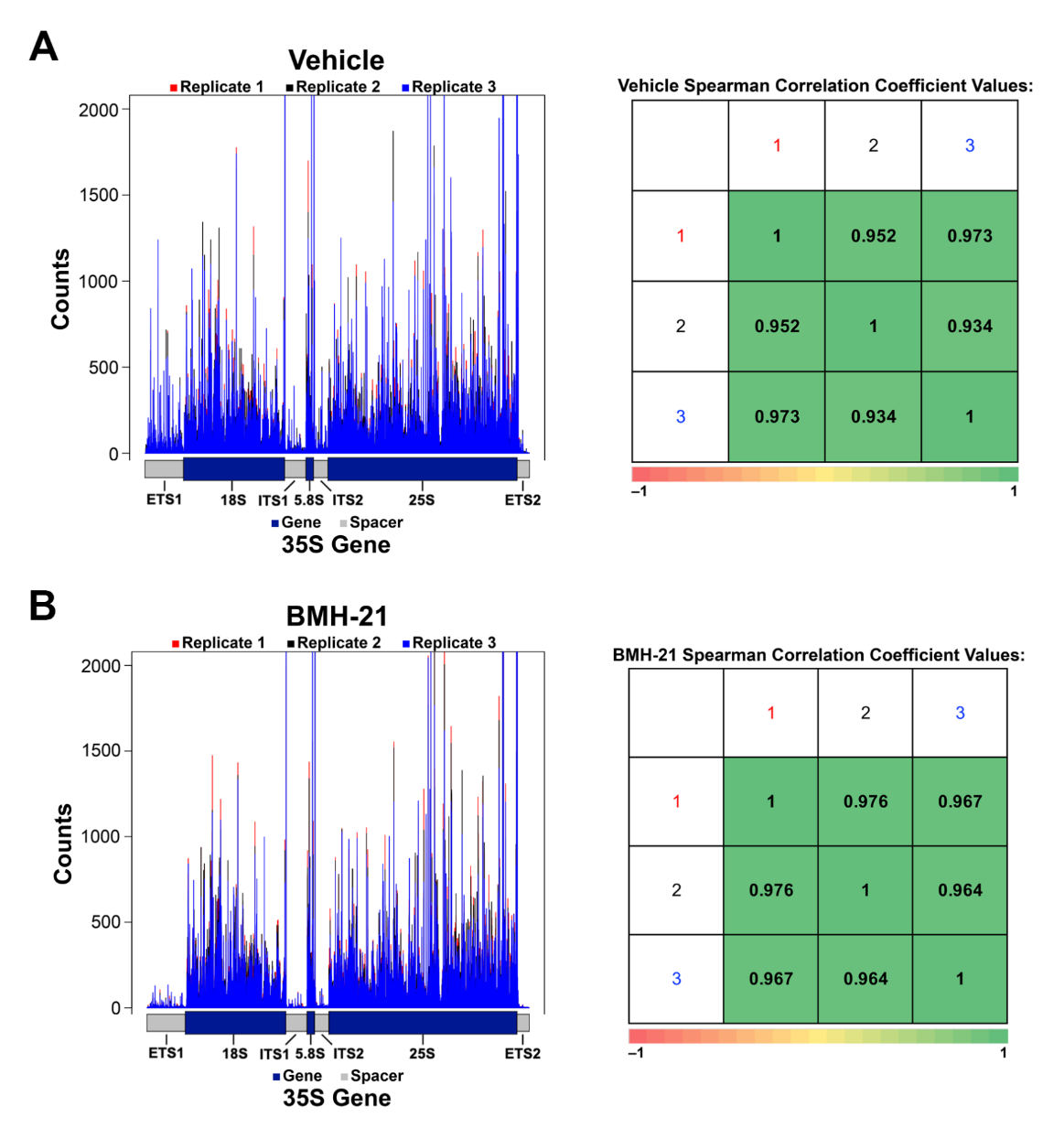
**

**Supplemental Figure S5. NET-seq experiments with a treatment time of 16 minutes are reproducible.**

Histograms (left panels) and Spearman correlation coefficient values (right panels) were generated for vehicle-treated (A) and BMH-21-treated (B) yeast. These experiments were performed in biological triplicate, and cells were treated with either vehicle buffer or BMH-21 for 15 minutes prior to an approximately 1-minute harvest.

**
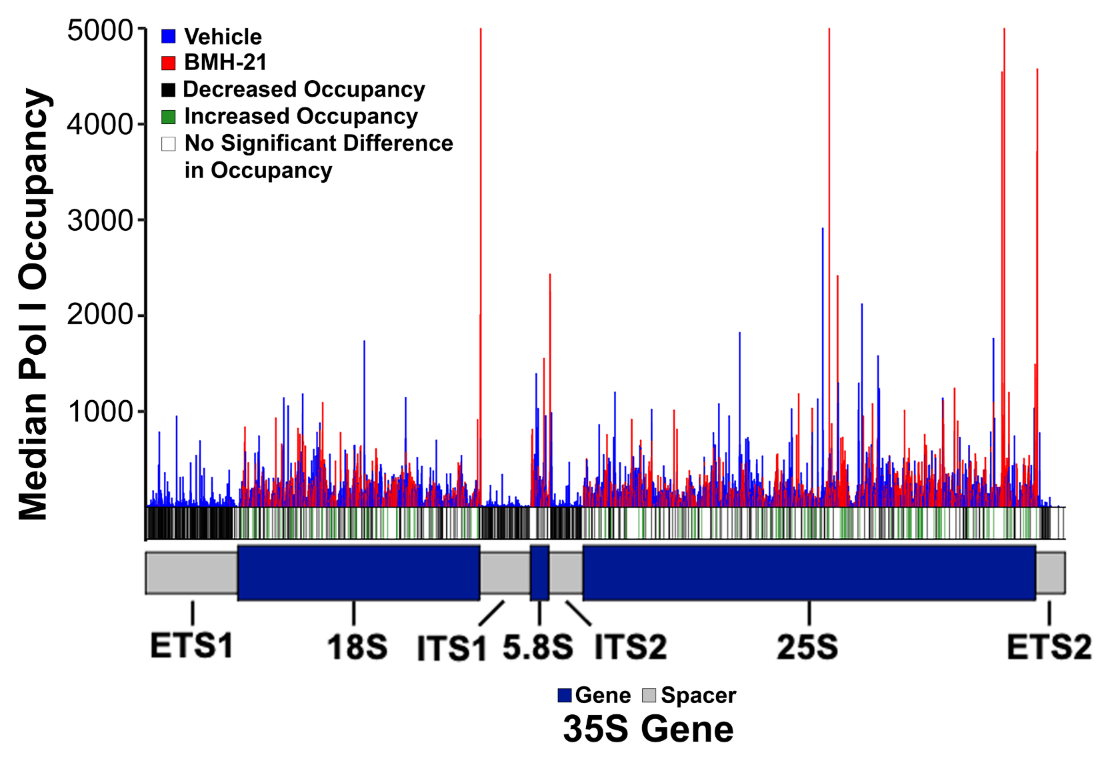
**

**Supplemental Figure S6. 16 minutes of BMH-21 treatment reduces Pol I occupancy.**

The median Pol I occupancy was determined and graphed from the libraries plotted in Figure S5. At each position, a t-test was performed to determine if there was a significant difference (*p* < 0.05) in occupancy. These differences are indicated in the bar below the histogram, with either green (increased occupancy), black (decreased occupancy) or white (no significant change) for the BMH-21-treated libraries vs. vehicle-treated.

**
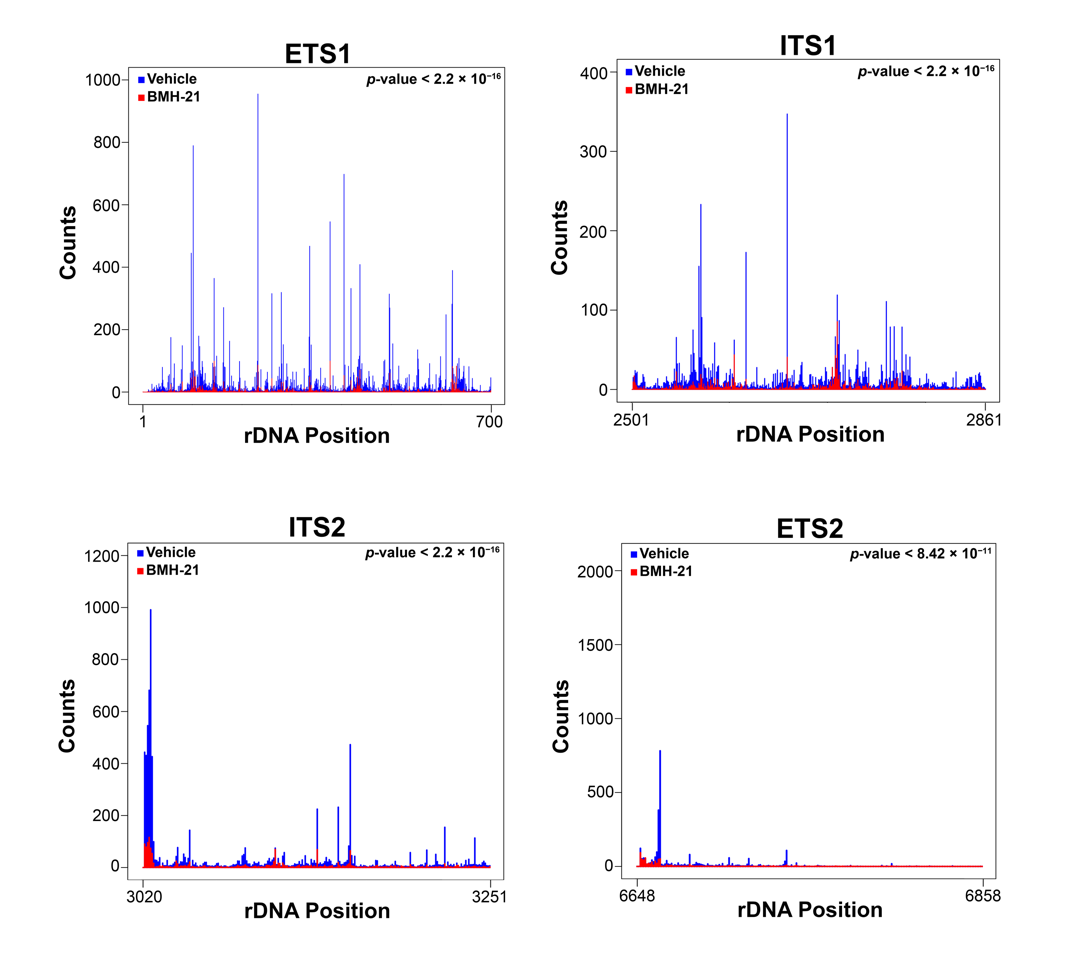
**

**Supplemental Figure S7. BMH-21 treatment reduces Pol I occupancy in the spacer regions after 16 minutes.**

The median occupancy for the vehicle-treated and BMH-21-treated libraries was plotted for the four spacer regions only (ETS1, ITS1, ITS2, and ETS2). The K-S test was run to determine whether the distribution patterns between treatment groups were significantly different, and these values are included in the inset at the top right corner of each graph.

**
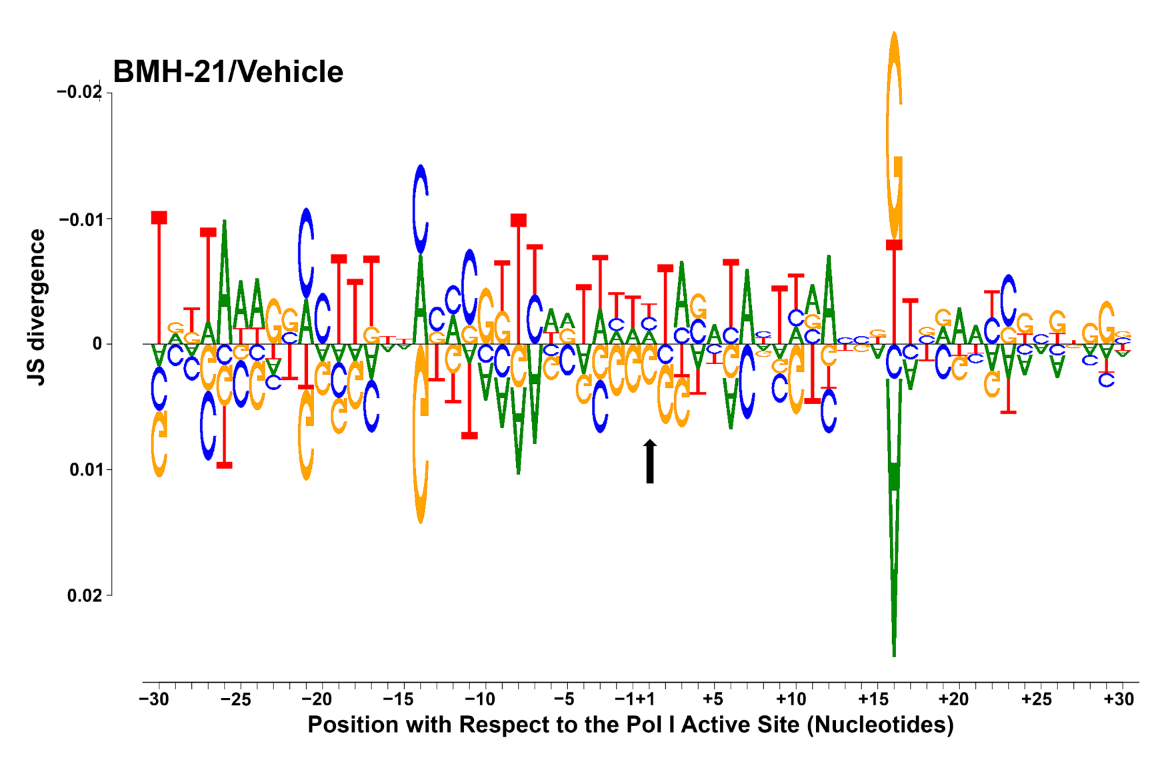
**

**Supplemental Figure S8. Pol I is repositioned on the rDNA template after 16 minutes of BMH-21 treatment.**

The software program DiffLogo was used to generate a DiffLogo of the sequence enrichments for the top 2.5% occupied positions in the spacer regions only for the BMH-21-treated (shown on top) and the vehicle-treated (shown on the bottom) libraries. The arrow indicates the last incorporated nucleotide into the RNA transcript, and these data are oriented with respect to the active site of Pol I.

| **Sample** | **Forward** | **Reverse** |
| --- | --- | --- |
| V1 | CAAGCAGAAGACGGCATACGAGATtcctctacTCCGACGATCATTGATGGTGCC | AATGATACGGCGACCACCGAGATCTACACtagatcgcCGTCTCTTCTGCGGATGACTCG |
| V2 | CAAGCAGAAGACGGCATACGAGATtgcctcttTCCGACGATCATTGATGGTGCC | AATGATACGGCGACCACCGAGATCTACACtagatcgcCGTCTCTTCTGCGGATGACTCG |
| V3 | CAAGCAGAAGACGGCATACGAGATcagcctcgTCCGACGATCATTGATGGTGCC | AATGATACGGCGACCACCGAGATCTACACtagatcgcCGTCTCTTCTGCGGATGACTCG |
| B1 | CAAGCAGAAGACGGCATACGAGATagcgtagcTCCGACGATCATTGATGGTGCC | AATGATACGGCGACCACCGAGATCTACACtagatcgcCGTCTCTTCTGCGGATGACTCG |
| B2 | CAAGCAGAAGACGGCATACGAGATcctctctgTCCGACGATCATTGATGGTGCC | AATGATACGGCGACCACCGAGATCTACACtagatcgcCGTCTCTTCTGCGGATGACTCG |
| B3 | CAAGCAGAAGACGGCATACGAGATgtagagagTCCGACGATCATTGATGGTGCC | AATGATACGGCGACCACCGAGATCTACACtagatcgcCGTCTCTTCTGCGGATGACTCG |

**Supplemental Table S1. Library amplification primers for NET-seq.**

This table includes the forward and reverse primers for the library amplification step of NET-seq. Each sample is included, with V1-3 indicating the vehicle-treated samples, and B1-3 indicating the BMH-21-treated samples.

| **Software** | **Version** |
| --- | --- |
| fqtrim | 0.9.7 |
| cutadapt | 1.12 |
| FastQC | 0.11.4 |
| STAR | 2.7.1a |
| SAMTools | 1.6 |
| BEDTools | 2.26.0 |
| R | 4.0.2 |
| dplyr | 1.0.2 |
| plyr | 1.8.6 |
| ggplot2 | 3.3.2 |
| ggseqlogo | 0.1 |
| ggpubr | 0.2.5 |
| cowplot | 1.1.1 |
| matrixStats | 0.58.0 |
| hexbin | 1.28.1 |
| tweedie | 2.3.3 |
| statmod | 1.4.35 |
| magritter | 1.5 |
| scales | 1.1.1 |
| tidyr | 1.1.2 |
| seqinr | 3.6-1 |
| zoo | 1.8-8 |
| DiffLogo | 2.14.0 |
| rclone | 1.48.0 |

**Supplemental Table S2. Software versions used for NET-seq data analysis.**

Software packages and the versions used are included in this table.
